# Clinical Features and Influencing Factors for the Prognosis of Patients With Sudden Deafness

**DOI:** 10.3389/fneur.2022.905069

**Published:** 2022-06-02

**Authors:** Wei Lin, Gaoyun Xiong, Kailei Yan, Wumin Yu, Xiaoxing Xie, Ze Xiang, Jian Wu, Yanping Ge, Ying Wang

**Affiliations:** ^1^Department of Otolaryngology, Tongde Hospital of Zhejiang Province, Hangzhou, China; ^2^The Second Clinical Medical College, Zhejiang Chinese Medical University, Hangzhou, China; ^3^Basic Medical College, Zhejiang Chinese Medical University, Hangzhou, China; ^4^Zhejiang University School of Medicine, Hangzhou, China; ^5^Department of Clinical Laboratory, The Affiliated Suzhou Hospital of Nanjing Medical University, Suzhou Municipal Hospital, Gusu School, Nanjing Medical University, Suzhou, China; ^6^Department of Infection Management, The Affiliated Suzhou Hospital of Nanjing Medical University, Suzhou Municipal Hospital, Gusu School, Nanjing Medical University, Suzhou, China

**Keywords:** clinical characteristics, risk factors, treatment effects, prognosis, sudden deafness

## Abstract

**Backgrounds:**

Studies on risk factors influencing the prognosis of patients with sudden onset deafness are lacking.

**Methods:**

From March 2018 to March 2021, 500 patients, from the Tongde Hospital in Zhejiang Province, with sudden onset deafness were enrolled. We collected clinical information from the hospital medical records, including certain demographic characteristics, information related to sudden-onset deafness, and laboratory parameters. Univariate and multivariate analyses were performed to determine independent prognostic risk factors for patients with sudden deafness. Additionally, we also employed orthogonal partial least squares discriminant analysis (OPLS-DA) to analyze the data of these enrolled patients.

**Results:**

The baseline clinical characteristics of the enrolled patients were analyzed. Based on their prognoses, the included patients were divided into the overall effective and ineffective groups. Between these two groups, the univariate and multivariate analyses were performed. Age, type of hearing curve at the initial diagnosis, acute phase, and sudden deafness site were found to be independently associated with the prognoses of patients with sudden deafness (all *P* < 0.05). Through the OPLS-DA, the sudden deafness site was found to be an indicator with the highest predictive power.

**Conclusions:**

Age, type of hearing curve at the initial diagnosis, acute phase, and sudden deafness site were all independently correlated with the prognoses of patients with sudden deafness and, therefore, need to be emphasized.

## Introduction

Sudden deafness, also known as idiopathic sudden deafness, refers to sudden and unexplained sensorineural hearing loss (1). In recent times, the incidence of sudden deafness is on the rise (2). Although large sample epidemiological data are lacking, the incidence of sudden deafness has been increasing in China in recent years (3). The incidence of sudden deafness in the United States ranges from 5 to 27 people per 100,000, and approximately 66,000 new cases are reported annually (4). The incidence of sudden deafness in Japan was 27.5 persons per 100,000 in 2001, and it shows an increasing year-on-year trend (5). The incidence reported in the German guidelines for sudden deafness in 2004 was 20 per 100,000, which increased to 160–400 per 100,000 per year in 2011 (6).

The etiology and pathophysiological mechanism underlying sudden deafness remains unclear. Local and systemic factors may lead to sudden onset deafness (7, 8). Common causes include vascular diseases, viral infection, autoimmune diseases, and so on (9). Mental tension, pressure, mood swings, irregular life, and sleep disorders also underlie the main causes of sudden onset deafness (10, 11). Currently, the recognized pathogenesis includes inner ear vasospasm, stria vascular dysfunction, vascular embolism or thrombosis, membrane labyrinth hydrops, and hair cell injury (12, 13).

The clinical manifestations of patients with sudden onset deafness include sudden hearing loss, tinnitus, ear fullness, dizziness, hyperacusis, and paresthesia around the ear (14, 15). Some patients even face psychological symptoms, such as anxiety and sleep disorder, which affect their quality of life (16, 17). Nowadays, several prognostic factors may affect the prognoses of patients with sudden onset deafness. Several previous studies have focused on the factors influencing different diseases from multiple perspectives (18–20). Nevertheless, the research on risk factors influencing the prognosis of patients with sudden onset deafness is incomplete. Therefore, we examined the independent risk factors for the treatment of patients with sudden deafness in a clinical cohort to provide new directions and insights into the treatment and prognosis of sudden deafness.

## Patients and Methods

### Patients

From March 2018 to March 2021, 500 patients who met the inclusion criteria for sudden deafness were enrolled at the Tongde Hospital in Zhejiang Province. Inclusion criteria for sudden deafness were as follows (21): (1) sensorineural hearing loss occurred suddenly within 72 h, with hearing loss of ≥ 20 dBHL in at least two adjacent frequencies, mostly unilateral, and a few cases could occur bilaterally or sequentially; (2) no clear etiology was found, including systemic or local factors; (3) patients may be accompanied by tinnitus, ear fullness, and paresthesia around the ear; and (4) patients may be accompanied by dizziness, nausea, and vomiting. Exclusion criteria were as follows: (1) patients who did not meet the above inclusion criteria; (2) those with organic lesions of the middle ear; (3) cases of sudden deafness and pregnant women suffering from systemic diseases, such as hypertension and diabetes; and (4) patients considered with the possibility of the posterior cochlear space-occupying lesion as evidenced by magnetic resonance imaging (MRI) of the skull or middle inner ear, or computed tomography (CT) scan of the temporal bone.

The following treatment methods were adopted in this study: antiviral drug ribavirin liquid injection, glucocorticoid oral prednisone, nerve adenosine triphosphate (ATP) oral nutrition, low molecular dextran, B vitamins, and other comprehensive treatment regimens; simultaneous oxygen therapy during infusion therapy, children under the age of 14, according to the weight-adjusted doses. After treatment for 7 days, bone-air conduction pure tone audiometry results were reviewed and compared with those before treatment.

Based on the curative effect grading standard of sudden deafness, hearing curative effects were evaluated as follows (21): recover: the average hearing threshold of the damaged frequency was completely restored to normal, or reached the level of the healthy ear; significantly effective: the average hearing threshold of damaged frequencies improved by ≥ 30dB; effective: the average hearing threshold of damaged frequencies improved by ≥ 15dB; and ineffective: the average hearing threshold of damaged frequencies improved by <15dB. Recovery, significantly effective, and effective were considered as the overall efficacy of the treatment.

We collected clinical information from the hospital medical records, including certain demographic characteristics, information related to sudden deafness, and laboratory parameters. This study design was approved by the Ethics Committee of the Tongde Hospital, Zhejiang Province (approval number: KTSC2019132). Informed consents were obtained from patients or their families.

### Data Analysis

Statistical analyses were performed using GraphPad Prism 9 and SIMCA. Univariate and multivariate analyses were used to determine independent risk indicators for the prognoses of sudden onset deafness patients. Data for all samples were analyzed using the Statistical Software SIMCA (Version 14.1.0.2047, 64-bit, MKS Umetrics, Umea, Sweden) for orthogonal partial least squares discriminant analysis (OPLS-DA). *P* < 0.05 was considered statistically significant.

## Results

### Clinical Characteristics of Enrolled Patients

A total of 500 patients with sudden onset deafness were enrolled in our study. Table 1 shows the baseline clinical characteristics of these included patients. The average age was 40 years. The age distribution of patients ranged between 30 and 54 years. Of the 500 patients with sudden onset deafness, 261 were males and 239 were females. According to the types of the hearing curve, flat type deafness was present in 52 patients, low-mid frequency decline type deafness in 187 patients, high-mid frequency decline type deafness in 157 patients, and total deafness in 104 patients. A total of 385 patients were in the acute phase within 3 weeks; the remaining 115 patients were not. Notably, none of the patients in this cohort had a family or genetic history of sudden onset deafness.

**Table 1:** Clinical characteristics of enrolled patients with sudden deafness.

**Variables**	**Sudden deafness patients (*n* = 500)**
Age (year)	40.00 (30.00–54.00)
Male (%)	261 (52.20)
Females (%)	239 (47.80)
***Initially diagnosed hearing curve types***	
Flat type deafness (%)	52 (10.40)
Low-mid frequency decline type deafness (%)	187 (37.40)
High-mid frequency decline type deafness (%)	157 (31.40)
Total deafness (%)	104 (20.80)
Acute phase (%)	385 (77.00)
Non-Acute phase (%)	115 (23.00)
Total bilirubin (μmol/L)	12.40 (9.60–16.00)
Indirect bilirubin (μmol/L)	9.95 (7.70–12.80)
Total protein (g/L)	73.40 (69.72–76.90)
Albumin (g/L)	45.00 (42.30–47.20)
Family history (%)	0
Genetic history (%)	0
***Sudden deafness site***	
Unilateral (%)	380 (76.00)
Bilateral (%)	120 (24.00)
Vertigo (%)	79 (15.80)
Tinnitus (%)	380 (76.00)
Hyperbaric oxygen treatment (%)	312 (62.40)

All patients underwent laboratory tests. Based on the results of the patients' examination, the average level of total bilirubin level was 12.40 (9.60–16) μmol/L, and the level of indirect bilirubin level was 9.95 (7.70–12.80) μmol/L, the total protein level was 73.40 (69.72–76.90) g/L, and the albumin level was 45 (42.30–47.20) g/L. Approximately 76% of these patients had unilateral, while 24% had bilateral sudden onset deafness. Moreover, 79 patients developed vertigo. About 380 patients presented symptoms of tinnitus. A total of 312 patients received hyperbaric oxygen treatment, while the remaining did not.

### Univariate and Multivariate Analyses of the Prognostic Risk Factors for Patients With Sudden Deafness

To determine the prognostic risk factors for patients with sudden onset deafness, 117 patients in our cohort were divided into the recovery, significantly effective, effective, and ineffective groups according to the observed treatment effects. A total of 110 patients were in the recovery group, 45 in the significantly effective group, 45 in the effective group, and 323 in the ineffective group. Patients in the recovery, significantly effective, and effective groups were considered to show overall effective treatment. Therefore, univariate and multivariable analyses for the prognostic risk factors were performed between the overall effective and ineffective groups (Table 2). Through univariate analysis, age, type of hearing curve at the initial diagnosis, acute phase, total protein level, albumin level, sudden deafness site, and hyperbaric oxygen treatment were found to be the significant risk factors for poor prognosis of patients with sudden onset deafness. Subsequently, the multivariable analysis was performed for these risk factors, and age, type of hearing curve at the initial diagnosis, acute phase, and sudden deafness site were found to be independently associated with prognoses of patients with sudden deafness (all *P* < 0.05).

**Table 2:** Univariate and multivariate analysis in the overall effective and ineffective groups.

**Variables**	**Overall effective group** **(*n* = 177)**	**Ineffective group** **(*n* =323)**	***P***	**Univariate analysis**		**Multivariate analysis**	
				**OR (95% CI)**	***P***	**OR (95% CI)**	***P***
Age (year)	34.00 (26.00–44.00)	45.00 (33.00–59.00)	<0.001	1.044(1.029–1.058)	<0.001	1.037(1.020–1.053)	<0.001
Males (%)	99 (55.93)	162 (50.15)	0.216	0.793 (0.549–1.146)	0.217		
***Initially diagnosed hearing curve types***			<0.001	1.484 (1.209–1.821)	<0.001	1.325 (1.057–1.660)	0.015
Flat type deafness (%)	19 (10.73)	33 (10.22)					
Low-mid frequency decline type deafness (%)	101 (57.06)	86 (26.63)					
High-mid frequency decline type deafness (%)	22 (12.43)	135 (41.80)					
Total deafness (%)	35 (19.77)	69 (21.36)					
Acute phase (%)	164 (92.66)	221 (68.42)	<0.001	0.172 (0.093–0.317)	<0.001	0.224 (0.118–0.424)	<0.001
Total bilirubin (μmol/L)	12.50 (9.55–16.20)	12.40 (9.60–15.70)	0.596	1.006 (0.984–1.028)	0.620		
Indirect bilirubin (μmol/L)	9.90 (7.65–12.90)	10.00 (7.70–12.60)	0.703	0.998 (0.965–1.033)	0.927		
Total protein (g/L)	73.85 ± 5.38	72.81 ± 5.57	0.044	0.966 (0.934–0.999)	0.044		
Albumin (g/L)	45.50 (42.75–47.70)	44.60 (42.00–47.00)	0.012	0.934 (0.886–0.985)	0.012		
Vertigo (%)	21 (11.86)	58 (17.96)	0.074	1.626 (0.950–2.781)	0.076		
Tinnitus (%)	135 (76.27)	245 (75.85)	0.916	0.977 (0.636–1.502)	0.916		
***Sudden deafness site***			<0.001	5.202 (2.918–9.272)	<0.001	3.447(1.856–6.402)	<0.001
Unilateral (%)	162 (91.53)	218 (67.49)					
Bilateral (%)	15 (8.47)	105 (32.51)					
Hyperbaric oxygen treatment (%)	100 (56.50)	212 (65.63)	0.044	1.471 (1.010–2.141)	0.044		

### OPLS-DA

**Figure 1:**
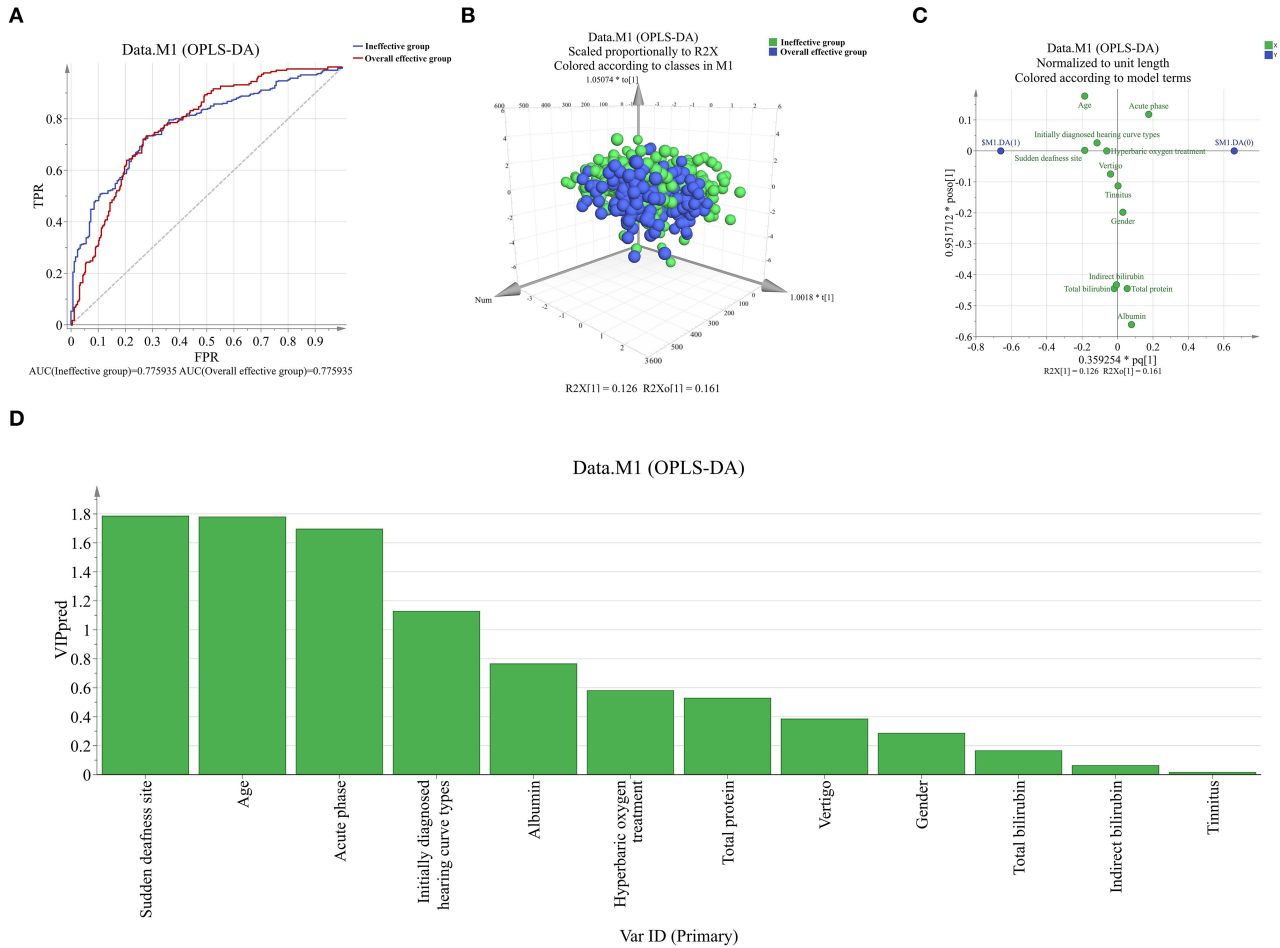
Orthogonal partial least squares discriminant analysis (OPLS-DA) assessed the ability of risk factors to predict the prognosis of patients with sudden deafness **(A,B)**. Through OPLS-DA, patients in the overall effective group could be unambiguously distinguished from patients in the ineffective group; **(C,D)**. Sudden deafness site was considered as the top 1 indicator with highest predictive capability.

To further rank and assess the risk factors for poor prognosis of patients with sudden deafness, the OPLS-DA method was used. According to the results of OPLS-DA, differences could be clearly distinguished between patients with sudden deafness in the overall effective and ineffective groups (Figures 1A,B). In addition, sudden deafness site was an indicator with the highest predictive power (Figures 1C,D).

## Discussion

Sudden deafness is a common emergency in otorhinolaryngology. The incidence ratio in males and females is similar, and, most frequently, comprises unilateral onset. Since the etiology and pathogenesis remain unclear, the clear clinical cause underlying sudden deafness is known in approximately 10% of these patients. It is difficult to evaluate the curative effects and prognoses due to the lack of standardized specific treatment and the presence of several influencing factors. Although sudden deafness shows a self-healing tendency, some patients recover to varying degrees. However, a poor prognosis is seen in some patients. Therefore, this present study focused on identifying the independent risk factors affecting the prognoses of patients with sudden deafness.

A total of 500 patients with sudden onset deafness with an average age of 40 years were enrolled. The age distribution ranged from 30 to 54 years. Among the patients enrolled in this study, 261 were males and 239 were females. According to the hearing curve types, 52 patients showed flat type deafness, 187 patients had low-mid frequency decline type deafness, 157 had high-mid frequency decline type deafness, and 104 patients had total deafness. Three hundred eighty five patients were in the acute phase within 3 weeks, while 115 were not. Notably, none of the patients in the cohort had a family or genetic history of sudden onset deafness. All included patients underwent laboratory tests. Based on these results, the total bilirubin level was 12.40 (9.60–16) μmol/L and the indirect bilirubin level was 9.95 (7.70–12.80) μmol/L. The level of total protein was 73.40 (69.72–76.90) g/L, and the level of albumin was 45 (42.30–47.20) g/L. In addition, 76% of these patients showed unilateral sudden deafness, while 24% had bilateral sudden deafness. A total of 79 patients developed vertigo, while 380 patients presented with symptoms of tinnitus. A total of 312 patients received hyperbaric oxygen treatment, while the remaining 188 patients did not receive it.

To determine the prognostic risk factors for patients with sudden deafness, 177 patients were divided into recovery, significantly effective, effective, and ineffective groups according to the outcomes of treatment received by patients with sudden deafness. Recovery, significantly effective, and effective were considered as overall effective. Univariate and multivariate analyses for risk factors were performed between the overall effective and ineffective groups. In univariate analysis, age, type of hearing curve at the initial diagnosis, acute phase, total protein level, albumin level, sudden deafness site, and hyperbaric oxygen treatment were identified as the risk factors for poor prognosis in patients with sudden deafness. Multivariate risk factor analysis was then performed. Based on these results, age, type of hearing curve at the initial diagnosis, acute phase, and sudden deafness site were all found to be independently associated with the prognosis of patients with sudden deafness (all *P* <.05). Among them, the older were the patients, the worse was the prognosis, mainly due to the vulnerability of the auditory system and other physical conditions that can exacerbate deafness (22). Additionally, sudden deafness site was also considered an important factor that influenced the prognosis of patients with sudden deafness, consistent with the results of several previous studies (23, 24). Therefore, these factors should be emphasized during the treatment of patients with sudden deafness, which would be conducive to their better treatment and prognosis.

To further rank and evaluate the risk factors affecting the prognosis of patients with sudden deafness, the OPLS-DA method was used for analysis. Based on the results, the difference between patients in the overall effective and ineffective groups could be distinguished. Moreover, sudden deafness site was considered to be an indicator with the highest predictive power. It was speculated that patients with bilateral sudden deafness may have a poorer prognosis than those with unilateral sudden deafness.

However, there are limitations to this study. First, the sample size of patients with sudden deafness needs to be increased to validate the above conclusions. Second, the patients in this study all came from the same hospital, which may contribute to certain regional deviations. Finally, the statistics of some clinical parameters in patients with sudden deafness were incomplete, and more clinical data are needed to evaluate the independent risk factors that may lead to poor prognoses of sudden onset deafness.

## Conclusion

In conclusion, age, type of hearing curve at the initial diagnosis, acute phase, and sudden deafness site were independently correlated with the prognosis of patients with sudden deafness (all *P* < 0.05). Notably, sudden deafness site was an indicator with the highest predictive power. These factors should be considered during the treatment of patients with sudden deafness. Our finding may thus provide better clinical guidance for the treatment and prognosis of patients with sudden deafness.

## Data Availability Statement

The raw data supporting the conclusions of this article will be made available by the authors, without undue reservation.

## Ethics Statement

The studies involving human participants were reviewed and approved by the Ethics Committee of the Tongde Hospital, Zhejiang Province (approval number: KTSC2019132). The patients/participants provided their written informed consent to participate in this study.

## Author Contributions

WL and GX: guarantor of this work. KY, WY, and XX: data collection. YG and YW: statistical analysis and manuscript writing. ZX and JW: critical revision of the manuscript. All authors contributed to the article and approved the submitted version.

## Funding

This study was funded by grants from Zhejiang basic public welfare research plan (LGC22H130001) and Zhejiang traditional Chinese medicine science and technology plan (20212A036).

## Conflict of Interest

The authors declare that the research was conducted in the absence of any commercial or financial relationships that could be construed as a potential conflict of interest.

## Publisher's Note

All claims expressed in this article are solely those of the authors and do not necessarily represent those of their affiliated organizations, or those of the publisher, the editors and the reviewers. Any product that may be evaluated in this article, or claim that may be made by its manufacturer, is not guaranteed or endorsed by the publisher.

## References

[1] Young Y-H. Contemporary review of the causes and differential diagnosis of sudden sensorineural hearing loss. Int J Audiol. (2020) 59:243–53. 10.1080/14992027.2019.1689432 31714154

[2] World Health Organization. Addressing the Rising Prevalence of Hearing Loss. Geneva: World Health Organization. (2018). Available online at: https://apps.who.int/iris/handle/10665/260336

[3] Lai D Zhong J Lu T. Pu J-M. Patient education in sudden sensorineural hearing loss: knowledge, attitude/belief, and practice findings among otolaryngologists and otologists in China Patient Educ Couns. (2019) 102:93–8. 10.1016/j.pec.2018.08.022 30146406

[4] Chandrasekhar SS Tsai Do BS Schwartz SR Bontempo LJ Faucett EA Finestone SA. Clinical practice guideline: sudden hearing loss (update). Otolaryngol Head Neck Surg. (2019) 161(Suppl. 1):S1–45. 10.1177/0194599819859883 31369359

[5] Alexander TH Harris JP. Incidence of sudden sensorineural hearing loss. Otol Neurotol. (2013) 34:1586–9. 10.1097/MAO.0000000000000222 24232060

[6] Michel O. The revised version of the german guidelines “Sudden Idiopathic Sensorineural Hearing Loss.” *Laryngorhinootolog*. (2011) 90:290–3. 10.1055/s-0031-1273721 21560090

[7] Suckfüll M. Perspectives on the pathophysiology and treatment of sudden idiopathic sensorineural hearing loss. Dtsch Arztebl Int. (2009) 106:669. 10.3238/arztebl.2009.0669 19946432 PMC2780011

[8] Sun XM Zhuang SM Xiao ZW Luo JQ Long Z Lan LC. Autoimmune thyroiditis in patients with sudden sensorineural hearing loss. Laryngoscope Investig Otolaryngol. (2022) 7:571–7. 10.1002/lio2.755 35434320 PMC9008166

[9] Hung W-C Lin K-Y Cheng P-W Young Y-H. Sudden deafness: a comparison between age groups. Int J Audiol. (2021) 60:911–6. 10.1080/14992027.2021.1900611 33752575

[10] Jin L Fan K Tan S Liu S Wang Y Yu S. Analysis of the characteristics of outpatient and emergency diseases in the department of otolaryngology during the “Covid-19” pandemic. Sci Prog. (2021) 104:00368504211036319. 10.1177/00368504211036319 34323155 PMC10358545

[11] Stachler RJ Chandrasekhar SS Archer SM Rosenfeld RM Schwartz SR Barrs DM. Clinical practice guideline: sudden hearing loss. Otolaryngol Head Neck Surg. (2012) 146(Suppl. 3):S1–35. 10.1177/0194599819859885 22383545

[12] Chen J He J Luo J Zhong S. Association of aenac P. Ala663thr gene polymorphism with sudden sensorineural hearing loss. Front Genetics. (2021) 12:659517. 10.3389/fgene.2021.659517 35024042 PMC8744410

[13] Acipayam H Koçak HE Elbistanli MS. Sudden sensorineural hearing loss. Excursus Hearing Loss. (2018):71.

[14] Conte G Di Berardino F Mastrapasqua RF Casale S Scola E Capaccio P. Prognostic value of early magnetic resonance imaging patterns in sudden hearing loss. Audiol Neurootol. (2022) 27:64–74. 10.1159/000515153 33895732

[15] Fife TD Tourkevich R. Tinnitus, hyperacusis, otalgia, and hearing loss. Continuum (Minneap Minn). (2021) 27:491–525. 10.1212/CON.0000000000000961 34351116

[16] Yuan Y Wang H Chen Q Xie C Li H Lin L. Illness experience and coping styles of young and middle-aged patients with sudden sensorineural hearing loss: a qualitative study. BMC Health Serv Res. (2021) 21:1–10. 10.1186/s12913-021-06763-z 34315453 PMC8314487

[17] Sun B Liu L Ren X Wang Z. Psychological state of patients with sudden deafness and the effect of psychological intervention on recovery. J Int Med Res. (2020) 48:0300060520957536. 10.1177/0300060520957536 32967513 PMC7521050

[18] Wu J Guo Y Lu X Huang F Lv F Wei D. Th1/Th2 Cells and associated cytokines in acute hepatitis E and related acute liver failure. J Immunol Res. (2020) 2020:1–8. 10.1155/2020/6027361 33294465 PMC7691005

[19] Wang L Wu J Song S Chen H Hu Y Xu B. Plasma exosome-derived sentrin sumo-specific protease 1: a prognostic biomarker in patients with osteosarcoma. Front Oncol. (2021) 11:296. 10.3389/fonc.2021.625109 33791211 PMC8006461

[20] Wu J Shi C Sheng X Xu Y Zhang J Zhao X. Prognostic nomogram for patients with hepatitis E virus-related acute liver failure: a multicenter study in China. J Clin Transl Hepatol. (2021) 9:828. 10.14218/JCTH.2020.00117 34966646 PMC8666371

[21] Chinese M. Guideline of diagnosis and treatment of sudden deafness. Zhonghua Er Bi Yan Hou Tou Jing Wai Ke Za Zhi. (2015) 50:443–7. 26695792

[22] Golmohammadi R Darvishi E. The combined effects of occupational exposure to noise and other risk factors– a systematic review. Noise Health. (2019) 21:125. 10.4103/nah.NAH_4_18 32719300 PMC7650855

[23] Oh J-H Park K Lee SJ Shin YR Choung Y-H. Bilateral vs. unilateral sudden sensorineural hearing loss. Otolaryngol Head Neck Surg. (2007) 136:87–91. 10.1016/j.otohns.2006.05.015 17210340

[24] Metrailer AM Babu SC. Management of sudden sensorineural hearing loss. Curr Opin Otolaryngol Head Neck Surg. (2016) 24:403–6. 10.1097/MOO.0000000000000287 27379548

